# Chronic Lithium Treatment Enhances the Number of Quiescent Neural Progenitors but Not the Number of DCX-Positive Immature Neurons

**DOI:** 10.1093/ijnp/pyv003

**Published:** 2015-03-06

**Authors:** N. Kara, S. Narayanan, R. H. Belmaker, H. Einat, V. A. Vaidya, G. Agam

**Affiliations:** Department of Clinical Biochemistry and Pharmacology (Ms Kara and Dr Agam), and Psychiatry Research Unit (Ms Kara and Drs Belmaker and Agam), Faculty of Health Sciences, Ben-Gurion University of the Negev, Beer-Sheva, Israel; Mental Health Center, Beer-Sheva, Israel (Ms Kara and Dr Agam); Tel Aviv-Yaffo Academic College, Tel Aviv-Yaffo, Israel (Ms Kara and Dr Einat); Tata Institute of Fundamental Research, Mumbai, India (Ms Narayanan and Dr Vaidya); University of Minnesota, Minneapolis, Minnesota (Dr Einat).

**Keywords:** immature neurons, lithium, Nestin-GFP transgenic mice, neurogenesis, quiescent neural progenitor cells

## Abstract

**Background:**

The term adult neurogenesis constitutes a series of developmental steps including the birth, survival, differentiation, maturation, and even death of newborn progenitor cells within neurogenic niches. Within the hippocampus progenitors reside in the neurogenic niche of the subgranular zone in the dentate gyrus subfield. At the different stages, designated type-I, type-IIa, type-IIb, type-III, and granule cell neurons, the cells express a series of markers enabling their identification and visualization. Lithium has been shown to increase hippocampal cell proliferation in the subgranular zone of the hippocampal dentate gyrus subfield of adult rodents and to stimulate the proliferation of hippocampal progenitor cells in vitro, but data regarding lithium’s ability to increase neuronal differentiation and survival is equivocal.

**Methods:**

To clarify the effect of lithium on adult hippocampal neurogenesis, we identified the effect of chronic lithium treatment on distinct stages of hippocampal progenitor development using adult Nestin-green fluorescent protein transgenic mice and immunofluorescent techniques.

**Results:**

The present observations confirm that lithium targets the initial stages of progenitor development enhancing the turnover of quiescent neural progenitors/putative stem-cells, corroborating previous reports. However, the enhanced quiescent neural progenitor-turnover does not translate into an increased number of immature neurons. We also observed a steep decline in the number of type-III immature neurons with complex tertiary-dendrites, suggesting that lithium alters the morphological maturation of newborn neurons.

**Conclusions:**

Our results do not corroborate previous reports of lithium-induced enhanced numbers of newly generated neurons.

## Introduction

**Figure 1.**
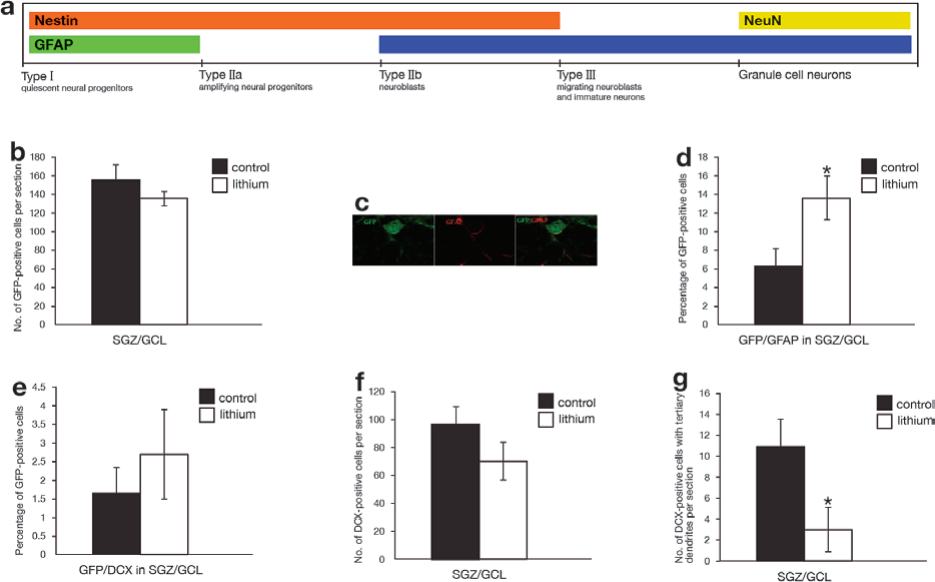
Effect of chronic lithium treatment on the number and percentage of different cell types in the hippocampus. (a) Lithium treatment significantly increased the percentage of Nestin-green fluorescent protein (GFP)/glial fibrillary acidic protein (GFAP) double-positive cells by about 2-fold. (b) Lithium treatment had no effect on the percentage of nestin-GFP positive cells. (c) Lithium treatment had no effect on the percentage of Nestin-GFP/ doublecortin (DCX) double-positive cells. (d) Lithium treatment had no effect on the number of DCX-positive cells. (e) Lithium treatment significantly decreased the number of type III cells exhibiting tertiary dendrites by about 75%. n=7/group in all experiments. Results are means±SEM. *P<.05 (Students *t* test).

The term adult neurogenesis constitutes a series of developmental steps including the birth, survival, differentiation, maturation, and even death of newborn progenitor cells within neurogenic niches. During the proliferation, fate choice, and eventual maturation of these progenitors, they transiently express a series of markers including nestin, glial fibrillary acidic protein (GFAP), doublecortin (DCX), and neuronal nuclei (NeuN), enabling the identification and visualization of adult progenitors at discrete stages of their development. Within the hippocampus, progenitors reside in the neurogenic niche of the subgranular zone (SGZ) in the dentate gyrus (DG) subfield. Slowly dividing radial glia-like progenitors (type-I cells/quiescent neural progenitors [QNPs]), expressing both nestin and GFAP, divide into transiently amplifying, rapidly dividing progenitors (type-IIa cells/amplifying neural progenitors) that express nestin but are no longer immunopositive for GFAP. Type-IIa cells differentiate into neuroblasts (type-IIb cells) expressing both nestin and DCX. Finally, migrating neuroblasts and immature neurons (type-III cells) express DCX and elaborate their dendritic and axonal arbors to mature into granule cell neurons expressing NeuN that integrate into functional hippocampal circuitry [Figure 1A (Ming and Song, 2011)].

Lithium has been shown to increase hippocampal cell proliferation in the SGZ of the hippocampal DG subfield of adult rodents and to stimulate the proliferation of hippocampal progenitor cells in vitro (Chen et al., 2000). However, existing data regarding lithium’s ability to increase neuronal differentiation and survival are equivocal. While some studies show that lithium induces differentiation towards a neuronal fate, as demonstrated by an increase in the number of NeuN-positive cells both in vitro and in vivo (Kim et al., 2004), other reports also indicate that despite the increase in the number of proliferating cells in the DG the percentage of NeuN-positive neurons generated remains unchanged (Chen et al., 2000; Li et al., 2011). In addition, a recent study suggests that the effects of lithium on progenitor turnover and survival are restricted to the ventral hippocampus and emerge only in animals concomitantly subjected to stress (O’Leary et al., 2012). The focus of the current study was to identify the effects of lithium on distinct stages of hippocampal progenitor development to clarify the effect of lithium on adult hippocampal neurogenesis.

Male Nestin-GFP transgenic mice (2 months) expressing transgenic green fluorescent protein (GFP) under the Nestin promoter (Yu et al., 2005), a kind gift of S.G. Kernie (Columbia University, NY), were bred and group-housed at the Tata Institute of Fundamental Research. Mice were supplied with food and water ad libitum and maintained on a 12-/12-hourlight/dark cycle at 24±1°C. The protocol was approved by the Tata Institute of Fundamental Research Animal Experimentation Ethics Committee and was in line with the NIH guide for the care and use of laboratory animals. Mice were treated with regular powdered food (RF) or lithium-supplemented RF for 14 days. The Li group received chow mixed with 0.2% LiCl for 5 days followed by 0.4% LiCl for 10 additional days (O’Brien et al., 2004). Mean plasma Li levels were 1.15 mM±0.38 (SD). Mice were sacrificed by transcardial perfusion with 4% paraformaldehyde; brains were removed and allowed to sink in 30% sucrose and frozen, and 40-µm sections generated on a sliding microtome. To detect GFP/GFAP/DCX-positive cells, a series of 4 hippocampal sections were selected for each brain and GFP/GFAP/DCX triple immunofluorescent staining was performed (Kapoor et al., 2012). To address the changes in adult hippocampal neurogenesis, immunohistochemistry with DCX, a microtubule-associated protein was performed. The number of GFP- and DCX-positive cells within the DG was quantified in 4 sections per animal using a Zeiss Axioskop at 40× in a blind manner. The morphological status of DCX-positive immature neurons was assessed by categorizing them as DCX+ cells with or without tertiary-dendrites. For quantification of triple immunofluorescence, 100 GFP-positive cells were assessed per animal to determine the number of nestin-GFP/GFAP and nestin/DCX double-positive cells using z-plane sectioning with 1-μm steps on a Zeiss LSM5 Exciter laser-scanning microscope to confirm colocalization.

Chronic lithium-treatment did not alter the total number of nestin-GFP-positive progenitors within the SGZ of the DG (Figure 1B); however, it significantly increased the percentage of Nestin-GFP/GFAP double-positive cells, indicating increased QNPs/type-I cell proliferation (Figure 1C-D). Lithium treatment did not affect the percentage of Nestin-GFP/DCX double-positive cells (Figure 1E), indicating a lack of effect on type-IIb (neuroblasts). Lithium treatment also had no effect on the number of DCX-positive cells per section, indicating that immature neuron number was unaltered (Figure 1F). However, the significant (approximately 75%) decline in the percentage of DCX-positive newborn neurons bearing complex tertiary dendrites suggests decreased morphological maturation (Figure 1G).

The results confirm that lithium targets the initial stages of progenitor development enhancing the turnover of QNP/putative stem cells, corroborating previous reports (Chen et al., 2000) and indicating lithium-induced enhanced progenitor proliferation assessed with bromodeoxyuridine (BrdU) within the SGZ. Our findings extend this observation, demonstrating that lithium targets the QNP stage similarly to electroconvulsive-seizure treatment, which enhances QNP turnover but distinctly from pharmacological antidepressants reported to influence ANPs turnover (Encinas et al., 2006; Segi-Nishida et al., 2008). However, it should be noted that Green and Nolan (2008) did not find lithium-induced change in BrdU-labelled cells under proliferation conditions in vitro (Green and Nolan, 2012). This discrepancy might stem from the difference between the in vivo and in vitro conditions. Similarly to Green and Nolan (2012), we found no effect of lithium on the total number of DCX-positive immature neurons, suggesting that the observed enhanced QNP turnover does not translate into an increased number of immature neurons. Strikingly, we also discerned a steep decline in the number of DCX-positive immature neurons with complex tertiary dendrites, suggesting that lithium alters newborn neurons morphological maturation. Our results do not corroborate previous reports of lithium-induced enhanced neurogenesis, which reported enhanced numbers of generated newborn neurons (Kim et al., 2004). However, both Chen et al. (2000) and Li et al. (2011) observed lithium-induced increased hippocampal cell proliferation but did not find a specific increase of neuronal differentiation. While lithium treatment increased the number of BrdU-positive cells in the adult DG, numbers of BrdU- and NeuN-double positive cells remained unchanged. Methodological issues might provide explanation for the different results: 1) in vitro vs in vivo lithium treatment; 2) different markers used to identify the neurogenesis stages; and 3) different regimes of in vivo lithium administration.

We further report that lithium significantly decreased the number of type-III cells exhibiting tertiary dendrites by 75%. Mature dendrites are more complex and have tertiary branches that extend to the outer molecular layer. This result is seemingly contradictory with the well-established lithium-induced elevated cell survival attributed to lithium’s inhibition of glycogen-synthase kinase-3. However, O’Leary et al. (2012) recently reported that chronic lithium treatment significantly decreased the survival of newly born cells in the DG. Newborn neurons have primary dendrites that further split off into secondary and tertiary dendritic branches. Hence, our result corroborates with lithium-induced decreased survival of newly born neurons. It is noteworthy that in the nematode *Caenorhabditis elegans* lithium treatment as well as mutations in the gene encoding for the enzyme inositol-monophosphatase (IMPase) inhibited by therapeutically relevant lithium concentrations, cause incorrect localization of neuronal synaptic proteins and behavioral defects, both rescued by expression of IMPase or by supplementation of inositol (the product of IMPase) (Tanizawa et al., 2006). These results suggest that IMPase is involved in the localization of synaptic components and normal behavior. It is tempting to speculate that our observation of the reduced number of type-III neurons with tertiary dendrites and the decreased survival of newborn neurons (O’Leary et al., 2012) following chronic lithium treatment are mediated by the drug’s inhibitory effect on IMPase.

Our results suggest that while lithium targets the QNP stage similarly to other fast-acting antidepressants, this does not translate into enhanced numbers of newborn neurons and rather appears to eventually evoke a decline in the morphological maturation of newborn neurons.

## Interest Statement

None.
